# Betaine Supplementation Attenuates S-Adenosylhomocysteine Hydrolase-Deficiency-Accelerated Atherosclerosis in Apolipoprotein E-Deficient Mice

**DOI:** 10.3390/nu14030718

**Published:** 2022-02-08

**Authors:** Xin Dai, Si Liu, Lokyu Cheng, Ting Huang, Honghui Guo, Dongliang Wang, Min Xia, Wenhua Ling, Yunjun Xiao

**Affiliations:** 1 Guangdong Provincial Key Laboratory of Digestive Cancer Research, The Seventh Affiliated Hospital, Sun Yat-sen University, Shenzhen 518107, China; daix23@mail.sysu.edu.cn (X.D.); liusi9135@163.com (S.L.); louisegurl@hotmail.com (L.C.); hting09@163.com (T.H.); 2 Department of Nutrition, School of Public Health, Guangdong Medical University, Dongguan 523808, China; guohh1999@gdmu.edu.cn; 3 Guangdong Provincial Key Laboratory of Food, Nutrition and Health, Department of Nutrition, School of Public Health, Sun Yat-sen University, Guangzhou 510080, China; wdliang@mail.sysu.edu.cn (D.W.); xiamin@mail.sysu.edu.cn (M.X.); lingwh@mail.sysu.edu.cn (W.L.)

**Keywords:** S-adenosylhomocysteine, inflammation, atherosclerosis, proliferation of smooth muscle cells, betaine

## Abstract

S-adenosylhomocysteine (SAH) is a risk factor of cardiovascular diseases and atherosclerosis. However, the causal association between SAH and atherosclerosis is still uncertain. In the present study, heterozygous SAH hydrolase (SAHH^+/−^) knockout mice were bred with apolipoprotein E-deficient mice to produce ApoE^−/−^/SAHH^+/−^ mice. At 8 weeks of age, these mice were fed on AIN-93G diets added with or without betaine (4 g betaine/100 g diet) for 8 weeks. Compared with ApoE^−/−^/SAHH^WT^ mice, SAHH deficiency caused an accumulation of plasma SAH concentration and a decrease in S-adenosylmethionine (SAM)/SAH ratio as well as plasma homocysteine levels. Betaine supplementation lowered SAH levels and increased SAM/SAH ratio and homocysteine levels in ApoE^−/−^/SAHH^+/−^ mice. Furthermore, SAHH deficiency promoted the development of atherosclerosis, which was reduced by betaine supplementation. The atheroprotective effects of betaine on SAHH-deficiency-promoted atherosclerosis were associated with inhibition of NFκB inflammation signaling pathway and inhibition of proliferation and migration of smooth muscle cells. In conclusion, our results suggest that betaine supplementation lowered plasma SAH levels and protected against SAHH-deficiency-promoted atherosclerosis through repressing inflammation and proliferation and migration of smooth muscle cells.

## 1. Introduction

Cardiovascular disease (CVD) is the world’s leading killer. Atherosclerosis is among the leading causes of CVD, myocardial infarction, and stroke worldwide. It is known that migration and proliferation of vascular smooth muscle cells (VSMCs) are key processes in the neointima formation that occurs during atherosclerosis [1]. Proliferating cell nuclear antigen (PCNA) is a class of proteins that is expressed in stages only in proliferating cells [2]. Detecting its expression can be used as an indicator to evaluate the state of cell proliferation. MAPKs activation, such as ERK1/2, are widely known as the main molecules responsible for initiating the signaling pathway involved in VSMCs proliferation, migration, and growth [3]. Furthermore, matrix metalloproteinases (MMPs), such as MMP2 and MMP9, degrade the extracellular matrix component and facilitate the migration of VSMCs [4]. In addition, atherosclerosis is a chronic inflammatory response to injury of the arterial wall. NF-κB has been proposed to be an integrator of many process that affect the formation of atherosclerosis plaques, and its activation represents a key mechanism that regulates various inflammatory and immune responses [5]. NF-κB can directly regulate a variety of genes overexpression, participating in the initiation and progression of atherosclerotic lesions, such as monocyte chemoattractant protein-1 (MCP-1), vascular cell adhesion molecule-1 (VCAM-1), and intercellular adhesion molecule-1 (ICAM-1), which result in the adhesion of monocytes to endothelial cells and proliferation and migration of VSMCs, and thus promote the occurrence and development of atherosclerosis [6,7]. S-adenosylhomocysteine (SAH), as the precursor of homocysteine (Hcy), is reversibly hydrolyzed to Hcy and adenosine by SAH hydrolase (SAHH). Many studies have shown that elevated levels of SAH are related to the risk of CVD and atherosclerosis [8,9,10,11,12]. However, the causal relationship between SAH and atherosclerosis is still uncertain. So far, it is unclear how to effectively reduce the plasma level of SAH and whether lowering plasma levels of SAH could prevent atherosclerosis.

A great many important clinical studies have reported that multivitamin supplementation (vitamin B_6_, B_12_, folic acid) can reduce plasma tHcy concentration but not the risk of vascular disease [13,14,15]. However, plasma levels of those vitamins are not correlated with plasma SAH levels [16]. Supplementation of them also cannot lower plasma SAH levels [17]. Betaine is a choline derivative that occurs naturally within cells of mammals as well as plants. It can be consumed through our diet of some vegetables, seafood, and staple food, of which the foods with the highest betaine concentration (mg/100 g) are wheat bran (1339), spinach (645), shrimp (218), and wheat bread (201) [18]. Betaine plays a dual role in human physiology as a methyl donor of methylation reactions and an osmotic regulator to regulate cell osmotic pressure, reduce stress response, and maintain intracellular balance [19]. Metabolism of betaine primarily takes place in the liver and kidney through the enzyme betaine-homocysteine methyltransferase (BHMT). This enzyme helps transfer the methyl group from betaine to the accumulated intermediate Hcy [20]. Hence, dietary sources of betaine or dietary betaine supplement could lower severe hyperhomocysteinemia or homocystinuria [21,22]. Furthermore, it has been reported that betaine could lower the increased intracellular concentration of SAH in isolated hepatocytes of ethanol-fed rats [23]. Importantly, the previous studies have shown that betaine intervention can alleviate atherosclerosis lesion in Apolipoprotein E-deficient (ApoE^−/−^) mice [24]. Furthermore, some studies have shown that low maternal betaine status during pregnancy was related to the increase of birth weight and obesity, and betaine supplementation during gestation led to lower fetal adiposity [25,26]. Higher betaine intake during lactation affected early intestinal microbes, improved glucose homeostasis, and resulted in lower adiposity throughout adulthood in offspring, which demonstrated an association between betaine and long-term metabolic health of offspring [27]. Therefore, this study will explore whether supplementation of betaine could lower plasma SAH and thereby reduce high-SAH-aggravated progression of atherosclerosis in ApoE^−/−^ mice.

## 2. Materials and Methods

### 2.1. Animal Procedures

Heterozygous SAHH gene (also called the Ahcy gene; NCBI Reference Sequence: NM_016661.3) knockout (SAHH^+/−^) mice, harboring genetic deletion of the region encoding the SAHH gene, were generated on a C57BL/6J background at Cyagen Biosciences using CRISPR/Cas9-based targeting and homology-directed repair [28]. Heterozygous SAHH knockout mice were bred with Apolipoprotein E-deficient mice to produce SAHH-deficient ApoE^−/−^ (ApoE^−/−^/SAHH^+/−^) mice. At 8 weeks of age, the mice were systematically divided into three groups according to similar mean body weights: ApoE^−/−^/SAHH^WT^ group, ApoE^−/−^/SAHH^+/−^ group, and ApoE^−/−^/SAHH^+/−^ + betaine group. Then mice were fed for 8 weeks with control AIN-93G diets for ApoE^−/−^/SAHH^WT^ group and ApoE^−/−^/SAHH^+/−^ group and experimental interventions with betaine (4 g betaine/100 g diet) supplement for ApoE^−/−^/SAHH^+/−^ + betaine group. The detail ingredients of the diets for three groups are shown in Table 1. The mice were housed in a temperature-controlled (24 °C) room with a 12-h light/dark cycle with free access to food and autoclaved water and kept in accordance with standard animal care requirements. The mice were weighed every week, and we calculated the consumption of water and feed every week. This study and all procedures were approved by the Sun Yat-sen University Animal Committees.

### 2.2. Biochemical Indexes Measurements

At the end of intervention, mice were anesthetized and exsanguinated through withdrawing the maximum amount of blood from the orbital vein. Fasting blood samples were collected into microtubes containing chilled EDTA and centrifuged immediately at 2000× *g* for 15 min; plasma was stored at −80 °C. Plasma lipids levels, including total cholesterol, triglycerides, and HDL cholesterol, were measured by enzymatic colorimetric assays using Triglycerides GPO reagent kits and commercial Cholesterol Reagent according to the manufacturer’s instructions (Nanjing Jian cheng Bioengineering Institute, Nanjing, China). Non-HDL cholesterol concentration was calculated by subtracting HDL cholesterol from total cholesterol. Plasma total homocysteine (tHcy) levels were assessed by high-performance liquid chromatography (HPLC) and fluorescence detection [29]. Plasma SAH and S-adenosylmethionine (SAM) levels were determined by stable-isotope dilution liquid chromatography-electrospray injection tandem mass spectrometry (HPLC-MS/MS) [30]. The validation of the method was described previously [31].

### 2.3. Assessment of Atherosclerotic Plaques by Oil Red O Staining

The hearts were stored in 10% formalin buffer solution at 4 °C and embedded in OCT compound and frozen. Then, 10-µm cryostat sections of the aortic sinus were cut from where the three aortic valves first appeared up to where the aortic valves disappeared and then were stained with Oil Red O [32]. The mean atherosclerotic plaque areas from five sections were calculated for each mouse. The plaque area was assessed by means of direct image capture from an RGB camera (JVCky-F30B) and quantified using Image Pro Plus software by an investigator blinded to the intervention of the mice.

### 2.4. Immunohistochemistry Staining

After blocking with 5% normal rabbit or goat serum, sections were treated with primary antibodies to CD68 (ab283654), PCNA (GB11010), SMA (GB13044), MCP-1 (ab214819), ICAM-1 (ab282575), and VCAM-1 (ab134047) overnight at 4 °C, followed by horseradish peroxidase-conjugated secondary antibodies for 1 h. Sections were incubated with diaminobenzidine (DAB) substrate and counterstained with hematoxylin for nuclear staining.

### 2.5. Measurements of VSMCs Proliferation and Migration

The proliferation of VSMCs was measured by the cell proliferation reagent WST-1 (Roche Molecular Biochemicals, Penzberg, Germany). The migration of VSMCs was performed by wound healing and transwell assay as described previously [33].

### 2.6. Western Blot

Total protein extraction kit (KeyGen Biotech, Nanjing, China) was used to extract total protein from the mouse aortas. Procedures were followed according to the manufacturer’s instruction. The proteins were concentrated on 6% SDS-PAGE gels and separated on 10–12% SDS-PAGE gels and then transferred on PVDF membranes (Merck Millipore, Billerica, MA, USA). Following incubation with a monoclonal antibody at 4°C overnight, the membranes were incubated with secondary antibodies at room temperature for 1 h. Finally, the data normalized by β-actin were scanned and assessed by using a ChemiDoc^TM^ Imaging System. The primary antibodies used in the present study were as follows: BHMT (ab211119), CD68 (ab283654), SMA (ab5694), PCNA (ab18197), MCP-1 (ab214819), ICAM-1 (ab282575), VCAM-1 (ab134047), p-ERK (ab223500), ERK (ab32537), p-NF-kB/p65 (ab194726), and NF-kB/p65 (ab32536).

### 2.7. Real-Time qPCR

RNA was reverse transcribed into cDNA using a GoScript™ Reverse Transcription System kit (Promega, Madison, WI, USA). Real-time quantitative Polymerase Chain Reaction (qPCR) was performed using GoTaq^®^ qPCR Master Mix (Promega) according to the manufacturer’s protocol. The entire reaction was carried out on a Bio-Rad CFX96TM Real-time PCR System. β-actin were used as internal references, and all experiments were repeated at least three times. Unless stated otherwise, relative expression levels of genes were calculated using the 2-ΔΔCt method. qPCR primers of MMP9 and MMP2 were designed using Primer Express software 3.0.

### 2.8. Statistical Analyses

Data are presented as means ± SEM, and each experiment was subjected to at least three biological replicates. Differences between two groups were determined by independent sample Student *t*-tests, and differences among multiple groups were determined by one-way ANOVA. A probability value < 0.05 denoted a significant difference.

## 3. Results

### 3.1. The Body Weight and Intake of Daily Food and Plasma Lipids Levels among the Three Groups

**Figure 1:**
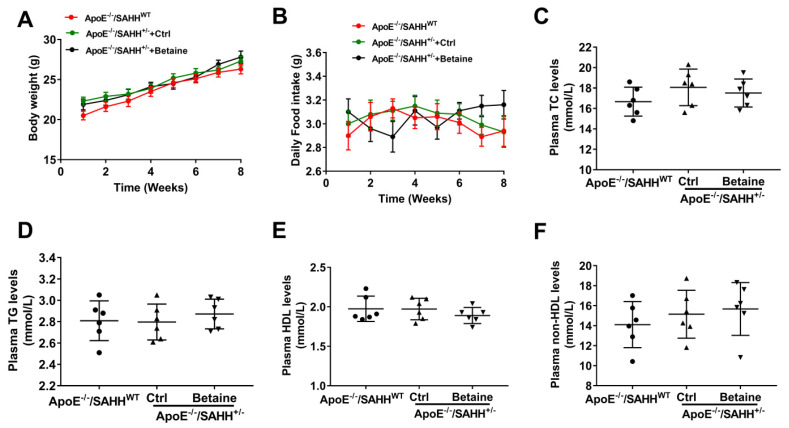
The body weight and daily intake of food and plasma lipids levels of ApoE^−/−^/SAHH^WT^ and ApoE^−/−^/SAHH^+/−^ mice with or without betaine supplementation. At 8 weeks of age, ApoE^−/−^/SAHH^+/−^ mice and their littermate ApoE^−/−^/SAHH^WT^ mice were fed with AIN-93G diets with or without added betaine (4 g betaine/100 g diet) for 8 weeks. (**A**) Change of body weights throughout the whole study among the three groups mice, *n* = 10 for each group. (**B**) Daily food intake of the three groups mice, *n* = 10 for each group. (**C**–**F**) The plasma lipids levels, *n* = 6 for each group. Values are mean ± SEM. *p* > 0.05 (determined by one-way ANOVA).

**Figure 2:**
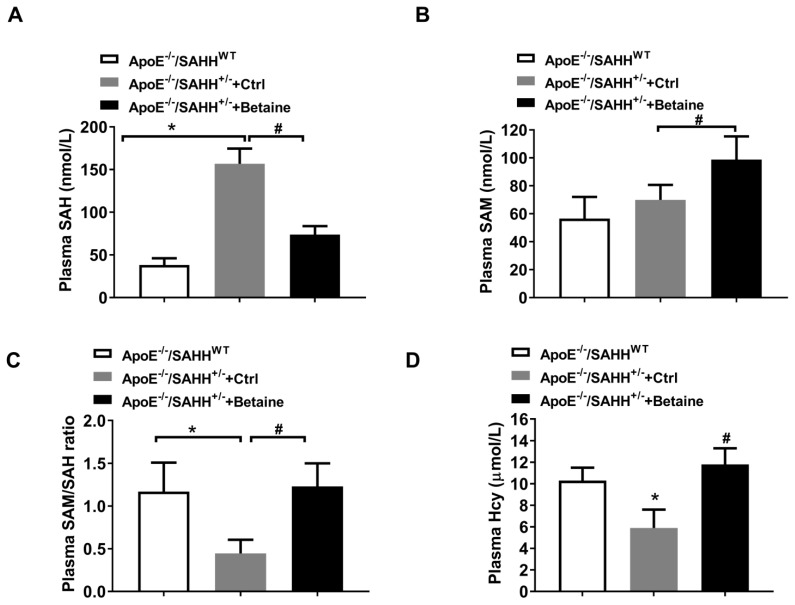
Betaine supplementation lowered SAHH deficiency-accumulated plasma SAH in ApoE^−/−^/SAHH^+/−^ mice. Plasma SAH (**A**) and SAM (**B**) levels were assessed by HPLC-MS/MS, *n* = 6 for each group. (**C**) The ratio of plasma SAM/SAH, *n* = 6 for each group. (**D**) The plasma levels of tHcy were measured by HPLC-FD, *n* = 6 for each group. Values are mean ± SEM; * *p* < 0.05 vs. WT; ^#^ *p* < 0.05 vs. Control (determined with one-way ANOVA).

To explore the role of betaine in SAH metabolism and atherosclerosis, we constructed an ApoE^−/−^ mouse model along with the heterozygous SAHH gene knockout (SAHH^+/−^), and then observed the effect of dietary betaine supplementation in SAHH-deficient ApoE^−/−^ mice. At 8 weeks of age, ApoE^−/−^/SAHH^+/−^ mice and their littermate ApoE^−/−^/SAHH^WT^ mice were fed with AIN-93G diets with or without added betaine (4 g betaine/100 g diet) for 8 weeks. There was no significant difference in the body weight and intake of daily food among the three groups of mice throughout of the whole study (Figure 1A,B). Additionally, no significant difference was observed in plasma lipids levels among the three groups (Figure 2C–F).

### 3.2. Betaine Supplementation Lowered SAHH-Deficiency-Accumulated SAH in ApoE^−/−^/SAHH^+/−^ Mice

Compared with the ApoE^−/−^/SAHH^WT^ group, plasma levels of SAH increased by more than three-fold in ApoE^−/−^/SAHH^+/−^ group, while plasma levels of SAH in the betaine-fed ApoE^−/−^/SAHH^+/−^ mice were notably lower than ApoE^−/−^/SAHH^+/−^ group (Figure 2A). SAHH gene knockout had no significant difference on plasma SAM levels, but the levels of plasma SAM were increased in betaine-treated mice (Figure 2B). Additionally, plasma SAM/SAH ratio in ApoE^−/−^/SAHH^+/−^ mice was markedly lower than that in the ApoE^−/−^/SAHH^WT^ group but was increased in betaine-fed ApoE^−/−^/SAHH^+/−^ group (Figure 2C). Plasma tHcy levels were significantly decreased in ApoE^−/−^/SAHH^+/−^ mice. However, betaine treatment significantly increased plasma tHcy levels (Figure 2D).

### 3.3. Betaine Supplementation Alleviated Atherosclerotic Lesions in ApoE^−/−^/SAHH^+/−^ Mice

**Figure 3:**
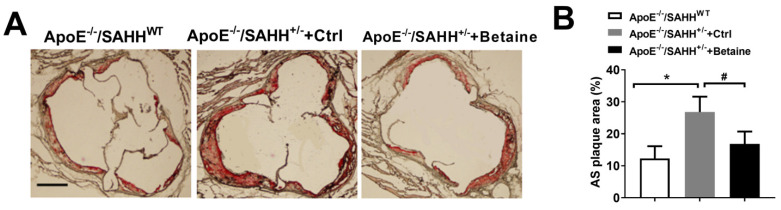
Betaine supplementation alleviated atherosclerotic lesions in ApoE^−/−^/SAHH^+/−^ mice. (**A**) Representative sections of aortic sinuses were stained by Oil Red O among the three groups of mice and (**B**) quantitative analyses of atherosclerotic lesions areas in aortic sinuses in three groups of mice, magnification 100×, *n* = 10 for each group. Values are mean ± SEM; * *p* < 0.05 vs. WT; ^#^ *p* < 0.05 vs. Control (determined with one-way ANOVA).

The atherosclerotic lesion area of aortic sinus was significantly increased by two-fold in ApoE^−/−^/SAHH^+/−^ group compared with ApoE^−/−^/SAHH^WT^ mice. However, betaine treatment significantly decreased the atherosclerotic plaque areas of aortic root in betaine-fed ApoE^−/−^/SAHH^+/−^ mice (Figure 3). The plaque area was positively correlated with SAH level but was inversely associated with SAM/SAH ratios, which is in agreement with the results in several human studies [31,34,35].

### 3.4. Betaine Supplementation Lowered SAHH-Deficiency-Accumulated SAH through Increasing Expression of BHMT

**Figure 4:**
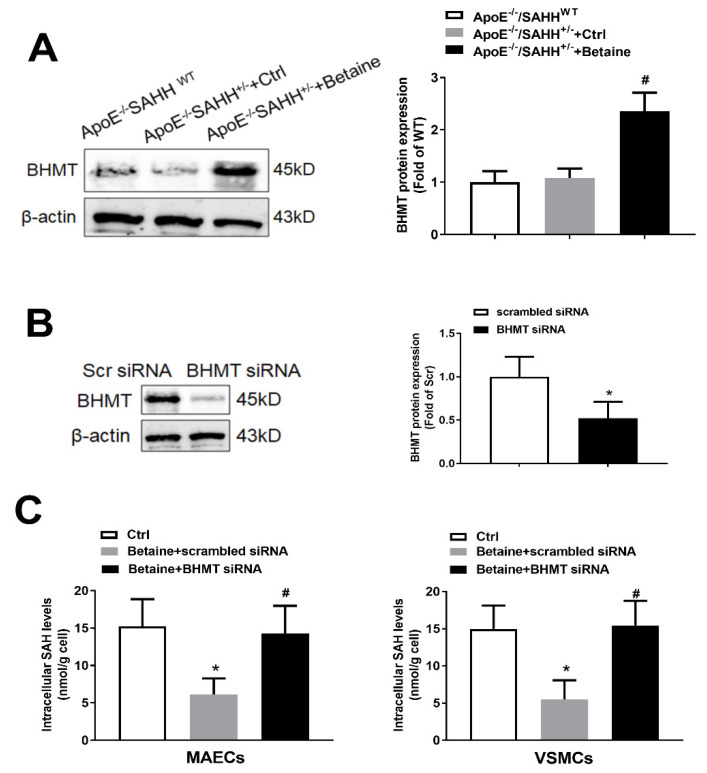
Betaine supplementation lowered SAH levels by increasing the expression of BHMT in ApoE^−/−^/SAHH^+/−^ mice. (**A**) Western blot analyses; the protein expression of betaine homocysteine S-methyltransferase (BHMT) in the aortas of the three groups, *n* = 6 for each group. (**B**) Mouse artery endothelial cells (MAECs) isolated from aortas of ApoE^−/−^/SAHH^+/−^ mice were transfected with BHMT siRNA or scrambled siRNA; the protein expression of BHMT was determined by Western blot; *n* = 3 for each group. (**C**) Intracellular SAH levels were measured by HPLC-MS/MS in MAECs and vascular smooth muscle cells (VSMCs) isolated from aortas of ApoE^−/−^/SAHH^+/−^ mice and treated with betaine (1 mmol/L) in the presence or absence of BHMT siRNA transfection, *n* = 3 for each group. Values are mean ± SEM; * *p* < 0.05 vs. WT; # *p* < 0.05 vs. Control or scrambled siRNA (determined with one-way ANOVA).

To investigate how betaine lowers the plasma SAH levels, we measured the protein expression of BHMT in the three groups. Although the protein expression of BHMT was not significantly changed between ApoE^−/−^/SAHH^+/−^ and ApoE^−/−^/SAHH^WT^ mice, betaine supplementation significantly increased BHMT expression in aortas of ApoE^−/−^/SAHH^+/−^ mice (Figure 4A). We further determined whether downregulation of BHMT could abolish the lowering effect of betaine on SAH levels. We used BHMT siRNA to transfect mouse artery endothelial cells (MAECs) isolated from aortas of ApoE^−/−^/SAHH^+/−^ mice, and the protein expression of BHMT was significantly reduced by BHMT siRNA transfection (Figure 4B). Knockdown of BHMT significantly abolished the lowering effect of betaine on intracellular SAH levels in MAECs. These effects were also observed in VSMCs isolated from aortas of ApoE^−/−^/SAHH^+/−^ mice (Figure 4C). Taken together, these results suggest that betaine supplementation can decrease SAH levels by increasing BHMT expression.

### 3.5. Quantitative and Phenotypic Differences in Atherosclerotic Plaque in ApoE^−/−^/SAHH^+/−^ Mice with or without Betaine Supplementation

**Figure 5:**
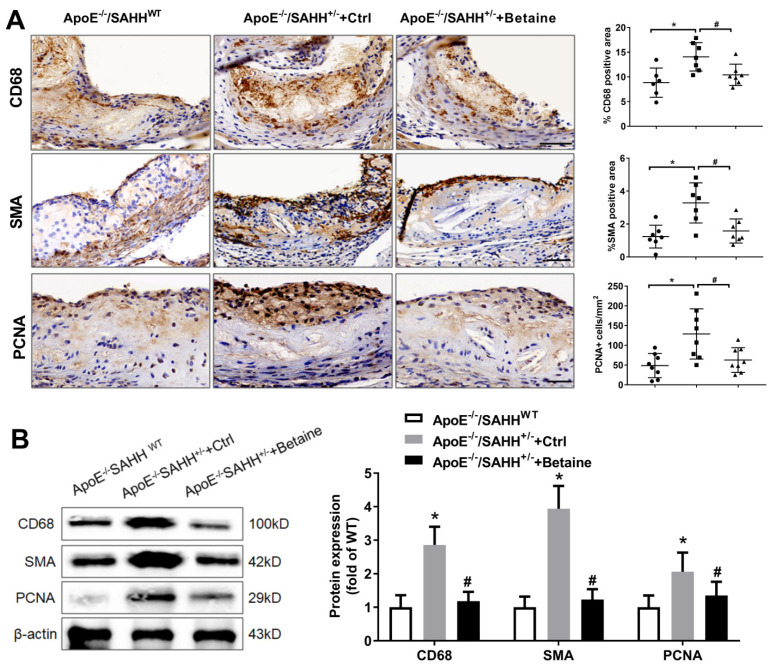
The effect of SAHH deficiency and betaine supplementation on atherosclerotic phenotype in ApoE^−/−^/SAHH^+/−^ mice. (**A**) Representative sections and quantitative analyses of aortic sinuses stained with antibodies specific for CD68^+^ macrophages, *n* = 6, 7, 7 for three groups, respectively; smooth muscle actin (SMA) for smooth muscle cells, *n* = 7 for each group; proliferating cells were stained with anti-proliferating cell nuclear antigen (PCNA) monoclonal antibody, *n* = 8 for each group. Scale bars = 100 μm, magnification 200×. (**B**) The protein expression of CD68, SMA, and PCNA were measured by Western blot in the aortas of the three groups, *n* = 6 for each group. Results are presented as mean ± SEM; * *p* < 0.05 vs. ApoE^−/−^/SAHH^WT^; ^#^ *p* < 0.05 vs. control group (determined by one-way ANOVA).

To determine the phenotypic changes of macrophage and smooth muscle cells in atherosclerotic lesions, we further performed immunohistochemistry and Western blot to detect the markers of macrophage and smooth muscle cells. After 8 weeks of pro-atherogenic diets, there was a three-fold increase in CD68^+^ cells in the atherosclerotic plaques of ApoE^−/−^/SAHH^+/−^ mice compared with ApoE^−/−^/SAHH^WT^ mice. Moreover, there was significantly increased smooth muscle cell content determined by SMA staining in these mice. More proliferating cells stained by PCNA were found in aortic sinus of ApoE^−/−^/SAHH^+/−^ mice than ApoE^−/−^/SAHH^WT^ mice. After 8 weeks of betaine supplementation, this treatment significantly reduced macrophage accumulation, smooth muscle cell content, and cell proliferation in ApoE^−/−^/SAHH^+/−^ mice (Figure 5). Taken together, these data suggest that SAHH-deficient ApoE^−/−^ mice have more active atherosclerotic lesions with more macrophages and smooth muscle cells, which could be attenuated by betaine supplementation.

### 3.6. Betaine Supplementation Attenuated the Levels of Inflammatory Markers in ApoE^−/−^/SAHH^+/−^ Mice

**Figure 6:**
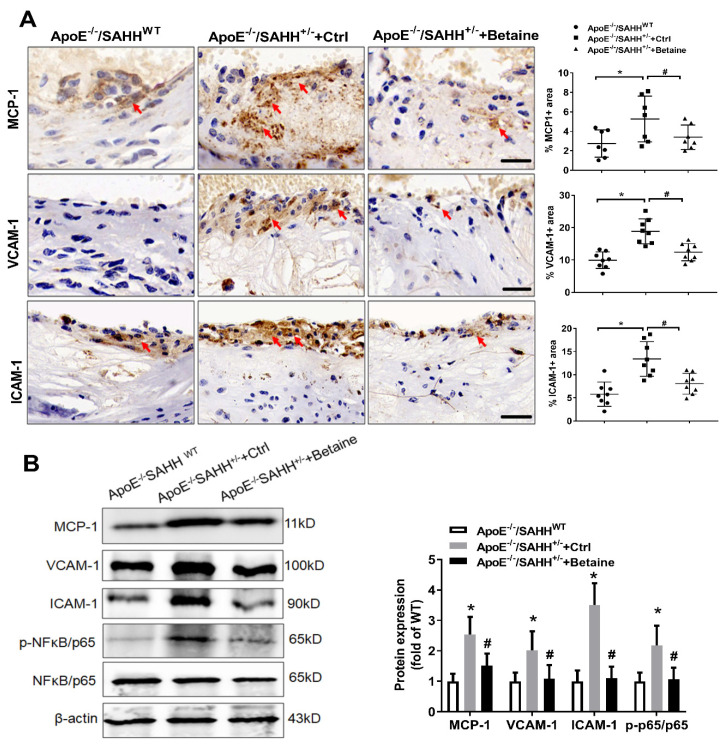
The effect of SAHH deficiency and betaine supplementation on expression of inflammatory markers in ApoE^−/−^/SAHH^+/−^ mice. (**A**) Representative photomicrographs and quantitative analyses of immunohistochemical staining for expression of MCP-1 (*n* = 7 for each group), VCAM-1 (*n* = 8 for each group), and ICAM-1 (*n* = 8 for each group) in atherosclerotic lesions of aortic sinuses from ApoE^−/−^/SAHH^+/−^ mice with or without betaine supplementation. Scale bars = 50 μm, magnification 400×. (**B**) The protein expression of MCP-1, VCAM-1, ICAM-1, and p-NFκB/p65 were measured by Western blot in the aortas of the three groups, *n* = 6 for each group. Results are presented as mean ± SEM; * *p* < 0.05 vs. ApoE^−/−^/SAHH^WT^; ^#^ *p* < 0.05 vs. Control; (determined by one-way ANOVA).

To determine the mechanisms by which betaine supplementation reduced SAHH deficiency-induced lesion development, we examined the effects on markers of inflammation. The expression of MCP-1 was markedly increased in plaques of ApoE^−/−^/SAHH^+/−^ mice; VCAM-1 and ICAM-1 were also elevated by about two-fold, whereas betaine supplementation significantly reduced the expression of VCAM-1, ICAM-1, and MCP-1 (Figure 6). To explore whether the effect of SAHH deficiency or betaine on inflammation is involved in NFκB signaling pathway, we examined the phosphorylation levels of NFκB/p65 in the three groups of mice. SAHH knockout significantly elevated phosphorylation levels of NFκB/p65, which were obviously repressed by betaine supplementation (Figure 6). Overall, this result indicates that the anti-atherogenic effect of betaine on SAHH-deficiency-promoted atherosclerosis may be associated with inhibition of NFκB inflammation signaling pathway.

### 3.7. Betaine Supplementation Inhibited the Proliferation and Migration of VSMCs Isolated from Aortas of ApoE^−/−^/SAHH^+/−^ Mice

**Figure 7:**
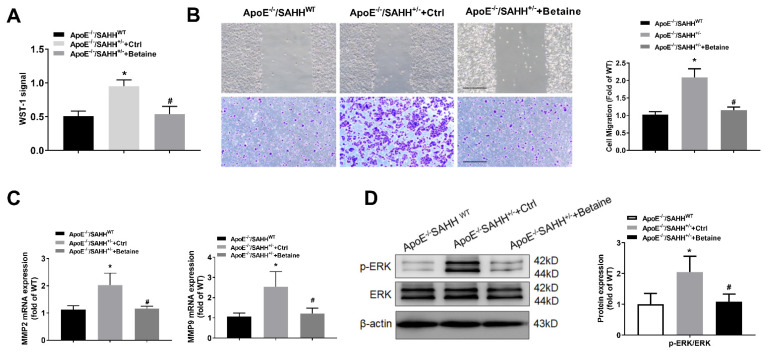
The effect of SAHH deficiency and betaine supplementation on the proliferation and migration of VSMCs isolated from aortas of ApoE^−/−^/SAHH^+/−^ mice. (**A**,**B**) The proliferation and migration of VSMCs isolated from aortas of ApoE^−/−^/SAHH^+/−^ mice were detected by WST-1 and wound healing and transwell assay; *n* = 3 independent experiments, magnification 100×. (**C**) The mRNA expression of MMP2 and MMP9 were measured by RT-qPCR in aortas of the three groups, *n* = 6 for each group; (**D**) The protein expression of p-ERK and total ERK were measured by Western blot in the aortas of the three groups, *n* = 6 for each group. Results are presented as mean ± SEM; * *p* < 0.05 vs. ApoE^−/−^/SAHH^WT^; ^#^ *p* < 0.05 vs. Control; (determined by one-way ANOVA).

Because the proliferation and migration of VSMCs are key events in the early formation of atherosclerosis, we further examined the effect of SAHH deficiency or betaine on the migration and proliferation of VSMCs in vitro. In agreement with our prevoius study, the migration and proliferation were significantly increased in VSMCs isolated from aortas of ApoE^−/−^/SAHH^+/−^ mice compared with those from ApoE^−/−^/SAHH^WT^ mice. Conversely, betaine treament inhibited the proliferation and migration of VSMCs isolated from aortas of ApoE^−/−^/SAHH^+/−^ mice (Figure 7A,B). To determine the expression of MMPs, which degrade the extracellular matrix component and facilitate the migration of VSMCs, we measured the mRNA levels of MMP2 and MMP9 by RT-qPCR in isolated VSMCs. SAHH knockdown increased the mRNA expression of MMP2 and MMP9, which were significantly lowered by betaine treament (Figure 7C). To determine whether the effect of SAHH deficiency or betaine on proliferation of VSMCs is involved in ERK signaling pathway, we examined the phosphorylation levels of ERK1/2 in the three groups of mice. SAHH knockout significantly increased phosphorylation levels of ERK1/2, which were obviously reduced by betaine supplementation (Figure 7D). Taken together, these results suggest that the anti-atherogenic effect of betaine on SAHH-deficiency-promoted atherosclerosis may be associated with inhibition of proliferation and migration of VSMCs.

## 4. Discussion

In the present study, we found partially SAHH deficiency caused an accumulation of plasma SAH levels and promoted atherosclerosis development, whereas betaine supplementation lowered SAH levels, restored the ratios of SAM/SAH, and reduced atherosclerotic lesions. The anti-atherogenic effect of betaine may be associated with inhibition of NFκB inflammation signaling pathway and proliferation and migration of VSMCs.

The first important finding is that lowering of plasma SAH levels by betaine supplementation could decrease the development of atherosclerosis. Others’ and our previous studies showed that increased plasma SAH levels were linked with increased risk of CVD and atherosclerosis [10,12,31,32,35]. However, the causal association between SAH and atherosclerosis is still unknown. Furthermore, endothelial function is impaired in SAHH^+/−^ mice [28], but the effect of SAHH knockout on atherosclerosis progression is also unclear. In the present work, we found that SAHH deficiency increased atherosclerotic lesions in ApoE^−/−^/SAHH^+/−^ mice. Importantly, betaine intervention can lower the increased plasma levels of SAH and reduce atherosclerosis in ApoE^−/−^/SAHH^+/−^ mice. Although previous study reported that betaine treatment could decrease the atherosclerotic lesions in ApoE^−/−^ mice [24], our results suggest that the lowering of plasma SAH may partially contribute to the anti-atherosclerotic effect of betaine in ApoE^−/−^/SAHH^+/−^ mice. Furthermore, the foods with the highest betaine content (mg/100 g) were wheat bran (1339), wheat germ (1241), spinach (645), pretzels (237), shrimp (218), and wheat bread (201) [18]. The dose of betaine added in the present study was 4 g betaine per 100 g food, which is approximately equivalent to wheat bran (299 g), wheat germ (322 g), spinach (620 g), pretzels (1687 g), shrimp (1835 g), or wheat bread (1990 g). Hence, this dose may be not usually achieved by betaine-rich foods but can be easily achieved by supplements.

Given that betaine mainly participates in the recycle metabolism of Hcy to methionine [20,36], which is then synthesized to SAM, our present study found that betaine treatment increased plasma SAM levels. However, inconsistent with some studies reporting the lowering effect of betaine on plasma tHcy levels [37,38,39], on the contrary, SAHH deficiency decreased plasma tHcy levels, whereas betaine treatment increased plasma tHcy levels in our study, suggesting that betaine may increase the degradation of SAH to Hcy and then accelerate its conversion to methionine and its metabolites, SAM. This point might be demonstrated by the finding that betaine supplementation could increase the expression of BHMT, which is a critical enzyme responsible for conversion of Hcy to methionine. However, downregulation of BHMT abolished the lowering effect of betaine on intracellular SAH. Furthermore, although moderate-or-severe high tHcy levels have been considered as a risk factor of CVD and atherosclerosis [40,41], physiological concentration of plasma tHcy less than 15μmol/L is not associated with increased risk of CVD and atherosclerosis [42]. On the contrary, in normal physiological conditions, transsulfuration pathway of plasma tHcy is the only source of cysteine, which is one component of glutathione (GSH) playing an important role in antioxidant function in body [20]. In the present study, knockdown of SAHH resulted in lower tHcy levels (about 5 μmol/L) and less than normal physiological concentration, whereas although betaine increased the tHcy levels, it was still in the physiological range. These different experiment conditions may explain the different effects of betaine on plasma tHcy levels.

Additionally, it was recently found that low SAM was related to increased risk of all-cause or cardiovascular mortality in patients with coronary artery disease [34]. Supplementation of SAM could inhibit proliferation and migration of VSMCs and reduce carotid intima thickness by reducing the inflammatory process and reducing endoplasmic reticulum and oxidative stresses [43] and prevent endothelial dysfunction by inducing heme oxygenase-1 expression [44]. Hence, elevation of SAM levels indirectly influenced by betaine may partially counteract the harmful effect of high SAH in atherosclerosis. Taken together, these evidences suggest that betaine supplementation may protect against SAHH-deficiency-promoted atherosclerosis by changing the metabolic balance between SAM and SAH.

Another important finding is that the anti-atherogenic effect of betaine may be related with inhibition of NFκB inflammation signaling pathway and proliferation and migration of VSMCs. In our previous finding, we reported that increased plasma levels of SAH promoted the proliferation and migration of VSMCs by activating oxidative stress-mediated ERK signaling pathway [33]. Consistently, our present study showed that SAHH deficiency caused an accumulation of plasma SAH levels and increased the proliferation and migration of VSMCs and phosphorylation levels of ERK, which were attenuated by betaine supplementation. Furthermore, macrophage and inflammation play pivotal roles in the development of atherosclerosis. It has been demonstrated that SAH could activate inflammation in endothelial cells by NFκB signaling pathway [45]. In agreement with this evidence, our findings showed that the effect of plasma SAH levels on the content of macrophage and expression of inflammatory markers was correlated with the activation of NFκB signaling pathway, which was also repressed by betaine supplementation in vivo.

## 5. Conclusions

In conclusion, our findings suggest that the atheroprotective effects of betaine on SAHH-deficiency-promoted atherosclerosis are associated with inhibition of NFκB inflammation signaling pathway and proliferation and migration of VSMCs. Our study provides novel evidence on the atheroprotective effects of betaine on SAH-associated atherosclerosis, indicating that dietary betaine supplementation might represent a new possible therapy strategy for SAH-associated atherosclerosis in the future.

## Figures and Tables

**Table 1:** The ingredients of the diets of three groups (g/kg diet).

Ingredient	ApoE^−/−^/SAHH^WT a^ (*n* = 10)	ApoE^−/−^/SAHH^+/− b^ (*n* = 10)	ApoE^−/−^/SAHH^+/−^ + Betaine (*n* = 10)
Cornstarch	397.486	397.486	397.486
Casein	200	200	200
Dextrinized cornstarch	132	132	132
Sucrose	100	100	100
Soybean oil	70	70	70
Fiber	50	50	50
Mineral mix (AIN-93G-MX) *	35	35	35
Vitamin mix (AIN-93G-VX) *	10	10	10
L-Cystine	3	3	3
Choline bitartrate	2.5	2.5	2.5
Betaine	—	—	40

^a^: Apolipoprotein E-deficient (ApoE^−/−^) mice with wild type of S-adenosylhomocysteine hydrolase (SAHH); ^b^: Apolipoprotein E-deficient (ApoE^−/−^) mice with heterozygous knockout of S-adenosylhomocysteine hydrolase (SAHH). * The detail composition of the AIN-93G-MX and AIN-93G-VX described previously by Reeves, P.G.; Nielsen, F.H.; Fahey, G.C. AIN-93 purified diets for laboratory rodents: final report of the American Institute of Nutrition Ad Hoc Writing Committee on the Reformulation of the AIN-76A Rodent Diet. *J. Nutr.***1993**, *123*, 1939–1951.

## Data Availability

The data presented in this study are available on request from the corresponding author.
